# Detecting 22q11.2 deletion in Chinese children with conotruncal heart defects and single nucleotide polymorphisms in the haploid *TBX1 *locus

**DOI:** 10.1186/1471-2350-12-169

**Published:** 2011-12-21

**Authors:** Yue-Juan Xu, Jian Wang, Rang Xu, Peng-Jun Zhao, Xi-Ke Wang, Heng-Juan Sun, Li-Ming Bao, Jie Shen, Qi-Hua Fu, Fen Li, Kun Sun

**Affiliations:** 1 Department of Pediatric Cardiology, Shanghai Children's Medical Center Affiliated to Shanghai Jiaotong University School of Medicine, Shanghai 200127, China; 2 Medical Laboratory, Shanghai Children's Medical Center Affiliated to Shanghai Jiaotong University School of Medicine, Shanghai 200127, China; 3 Medical Laboratory, Scientific Research Center, Xinhua Hospital Affiliated to Shanghai Jiaotong University School of Medicine, Shanghai 200092, China; 4 Medical Laboratory, Shanghai Children's Hospital Affiliated to Shanghai Jiaotong University School of Medicine, Shanghai 200040, China; 5 Division of Human Genetics, Cincinnati Children's Hospital Medical Center and University of Cincinnati College of Medicine, Cincinnati, Ohio 45229, USA; 6 Department of Pediatric Cardiology, Shanghai Children's Hospital Affiliated to Shanghai Jiaotong University School of Medicine, Shanghai 200040, China; 7 Department of Pediatric Cardiology, Xinhua Hospital Affiliated to Shanghai Jiaotong University School of Medicine, Shanghai 200092, China

## Abstract

**Background:**

Conotruncal heart defects (CTDs) are present in 75-85% of patients suffering from the 22q11.2 deletion syndrome. To date, no consistent phenotype has been consistently correlated with the 22q11.2 deletions. Genetic studies have implicated *TBX1 *as a critical gene in the pathogenesis of the syndrome. The aim of study was to determine the incidence of the 22q11.2 deletion in Chinese patients with CTDs and the possible mechanism for pathogenesis of CTDs.

**Methods:**

We enrolled 212 patients with CTDs and 139 unrelated healthy controls. Both karyotypic analysis and multiplex ligation-dependent probe amplification were performed for all CTDs patients. Fluorescence *in situ *hybridization was performed for the patients with genetic deletions and their relatives. The *TBX1 *gene was sequenced for all patients and healthy controls. The *χ*^2 ^and Fisher's exact test were used in the statistical analysis.

**Results:**

Thirteen of the 212 patients with CTDs (6.13%) were found to have the 22q11.2 deletion syndrome. Of the 13 cases, 11 presented with a hemizygous interstitial microdeletion from *CLTCL1 *to *LZTR1*; one presented with a regional deletion from *CLTCL1 *to *DRCR8*; and one presented with a regional deletion from *CDC45L *to *LZTR1*. There were eight sequence variants in the haploid *TBX1 *genes of the del22q11 CTDs patients. The frequency of one single nucleotide polymorphism (SNP) in the del22q11 patients was different from that of the non-del patients (*P *< 0.05), and the frequencies of two other SNPs were different between the non-del CTDs patients and controls (*P *< 0.05).

**Conclusions:**

CTDs, especially pulmonary atresia with ventricular septal defect and tetralogy of Fallot, are the most common disorders associated with the 22q11.2 deletion syndrome. Those patients with both CTDs and 22q11.2 deletion generally have a typical or atypical deletion region within the *TBX1 *gene. Our results indicate that *TBX1 *genetic variants may be associated with CTDs.

## Background

Congenital heart defects (CHDs) comprise a group of structural abnormalities with a combined incidence rate of approximately 1% of all live births. Of the cases of CHDs, 15-20% are conotruncal heart defects (CTDs) [1]. CTDs share the morphological architecture of the presence of ventricular outflow tract defects. They include tetralogy of Fallot (TOF), pulmonary atresia with ventricular septal defect (PA/VSD), double outlet of right ventricular (DORV), transposition of the great arteries (TGA), persistent truncus arteriosus (PTA) and interrupted aortic arch (IAA).

Although CTDs are generally considered complex, multifactorial disorders, a number of familial cases suggesting Mendelian inheritance have been described. The DiGeorge/Velo-cardio-facial syndrome (DGS/VCFS) is a genetic condition related to the 22q11.2 deletion, and 75-85% of the deletion patients present with a CTD. Reports originating from Western countries associate 12.8-17.8% of their CTD cases to the 22q11.2 deletion [2-5]. Most of the 22q11.2 deletions are sporadic in origin; however, approximately 10% of the deletions are inherited [6]. In total, 84-90% of the patients have a common region of deletion that is approximately 3 Mb deletion, which contains about 40 genes; while 7-14% present with a smaller, less common proximal ~1.5 Mb nested deletion, containing about 30 genes [7]. The deletions are reported to occur as a result of a non-allelic homologous recombination between the low-copy repeats (LCRs) in the 22q11.2 region [8,9]. A minority of patients have smaller overlapping and non-overlapping 22q11.2 deletions [10].

The *TBX1 *gene maps to the 1.5 Mb typically deleted region associated with DGS/VCFS and encodes a transcription factor of the T-box family known to play an important role in the regulation of developmental processes [11]. A *TBX1 *haploinsufficiency is thought to be responsible for many of the phenotypic traits of the DGS/VCFS. Mutations of the *TBX1 *gene have been detected in some patients featuring DGS/VCFS who are otherwise devoid of the 22q11.2 deletion [12-14].

Our previous screening for 22q11.2 deletions in 24 Chinese patients with CTDs by fluorescence *in situ *hybridization (FISH) showed a lower incidence rate (4.2%) of del22q11 than that usually reported (12.8-17.8%) [2-5,15]. In order to better understand the incidence of del22q11 in Chinese patients with CTDs, we carried out this current study in a new population of 212 patients.

## Methods

### Subjects

**Figure 1:**
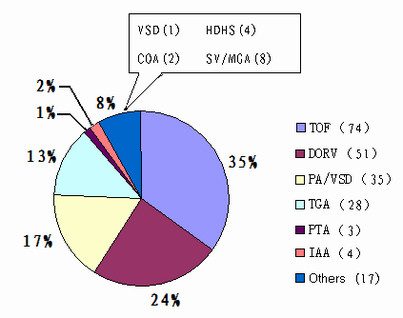
**Distribution of phenotypes of cardiac anomalies in patients with CTDs in this study**. Abbreviations: TOF: tetrology of Fallot; DORV: Double outlet of right ventricle; PA/VSD: pulmonary atresia/ventricular septal defect; TGA: transposition of the great arteries; PTA: persistent truncus arteriosus; IAA: interruption of aortic arch; VSD: ventricular septal defect; HDHS: hypoplastic right heart syndrome; COA: coarctation of aorta; SV/MGA: single ventricle/malposition of great arteries.

All assessments were undertaken with the approval of the Medical Ethics Committee of the Shanghai Children's Medical Center (SCMC). Patients were recruited prospectively from June 2008 to December 2009 at the SCMC. A total of 212 patients with CTDs were enrolled, of whom 92 were female and 120 male. The median age was 2.69 years old, and all of the patients were of the Han ethnicity. They included 74 TOF, 51 DORV, 35 PA/VSD, 28 TGA, 4 IAA and 3 PTA patients as well as 17 other cases of conotruncal malformations (Figure 1). Diagnoses were confirmed by transthoracic echocardiography and cardiac catheterization. When available, the surgical operative notes were reviewed. Extracardiac anomalies were also evaluated. Informed written parental consents were obtained for all of the patients participating in the study before blood samples were drawn at the time of catheterization.

Normal controls included 139 unrelated healthy Chinese children (57 female, 82 male; all ethnically Han) for detecting genetic variations at the *TBX1 *locus. The median age was 7.43 years old. The cardiac morphology of these controls was confirmed to be normal by transthoracic echocardiography at the SCMC. After obtaining informed written parental consent, a peripheral venous blood sample was collected into an anticoagulation tube with sodium citrate. Genomic DNA was extracted from peripheral lymphocytes using a QIAamp DNA Blood Mini Kit in keeping with the manufacturer's instructions (Qiagen, Duesseldorf, Germany).

### Cell culture and karyotyping

The karyotypes were examined using GTG-banding at a 400 band level. The lymphocytes obtained from the peripheral blood of the patients were cultured at 37°C with 5% CO_2_. The slide preparation for karyotyping was performed using the cultured peripheral blood cells. The G-banding was performed on these slides using a modification of Seabright's technique [16]. In each case, at least 20 banded metaphases with good chromosome separation were analyzed by experienced geneticists.

### Multiplex ligation-dependent probe amplification (MLPA)

**Figure 2:**
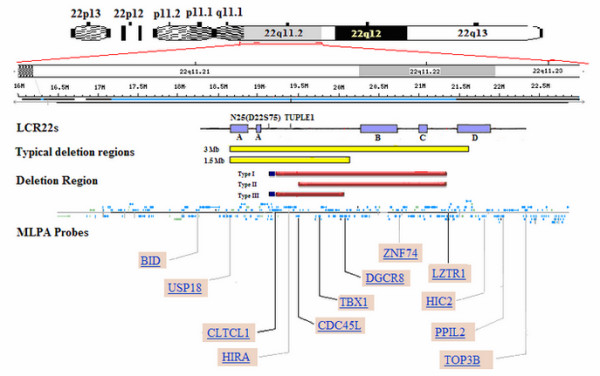
**Genomic map of the 22q11.2 region based on human genome overview page (Build 37.1)**. The relative positions of LCR22s and ranges of LCR22-A, -B, -C, and -D, as defined by Shaikh *et al*. [5], are depicted. The FISH probes *N25 *(*D22S75*) and *TUPLE1 *are marked on the LCR22s. The 3 Mb and 1.5 Mb typical deletion regions are shown in yellow. The MLPA probes were arranged according to the manufacturer's product information (MRC-Holland) and the position of the related gene in chromosome 22q11.2. The three types of deletion regions are delineated as Type I, Type II, and Type III, corresponding to the *N25*(*D22S75*) to *LZTR1, CDC45L *to *LZTR1*, and *N25*(*D22S75*) to *LZTR1*, respectively. The red lines indicate the deletion regions detected by MLPA analysis, whereas the blue lines designate the results of the FISH analysis.

Genomic DNA was extracted from peripheral lymphocytes using a QIAamp DNA Blood Mini Kit according to the manufacturer's instructions (Qiagen). For detection of the 22q11.2 deletion, we used a commercial SALSA P250-A1 MLPA-DiGeorge syndrome test kit (MRC-Holland, Amsterdam, The Netherlands). The P250-A1 MLPA probemix contains 48 probes for the frequent 22q11 deletion regions and the infrequent deletion regions. These probes are capable of distinguishing the most common types of deletion, 30 of which are related to the chromosomal region of 22q11. Five probes cover the Cat Eye Syndrome (CES) region, and another 14 fall on the most common DiGiorge deletion region. The 30 probes hybridize to a 7.3 Mb genomic region on chromosome 22q11. The most commonly deleted region starts at probe *CLTCL1*, downstream of LCR-A, and ends at probe *LZTR1*, upstream of LCR-D. The genomic distance between probes *CLTCL1 *and *LZTR1 *is approximately 2.2-2.5 Mb (Figure 2). The remaining 18 probes are located on 22q13, 10p15, 8p23, 4q34-qter, 9q34.3 and 17p13.3.

The MLPA reactions were performed following the manufacturer's instructions on all CTDs patients. After denaturation, hybridization, ligation and amplification, the polymerase chain reaction (PCR) products were sequenced by capillary electrophoresis using an ABI 3130 Genetic Analyzer (Applied Biosystems, Foster City, CA, USA). The raw data from electrophoresis were imported into the GeneMapper 4.0 software program (Applied Biosystems) and then analyzed using the Coffalyser MLPA DAT software package (MRC-Holland). After normalization, the data were compared to a synthetic control sample, which represents the median of all normal samples in each experiment. A threshold of signal intensity change of <0.75 was used to identify potential deletions, and a threshold of >1.33 was used to identify potential duplications. All samples with potential deletions or duplications were re-analyzed by MLPA.

### FISH

The 22q11.2 deletions detected in patients by MLPA were confirmed by FISH analysis. Commercially available locus specific probe kits N25 and TUPLE1 were purchased from Vysis (Downers Grove, IL, USA). The N25 kit contains the spectrum orange *N25 *(*D22S75*) probe for 22q11.2 and the spectrum green *ARSA *gene control probe for 22q13.3. The TUPLE1 kit contains the spectrum orange *TUPLE1 *(*HIRA*) probe at 22q11.2 and the spectrum green TelVysion 22q probe at 22q13.3.

The FISH probes *N25 *(*D22S75*) and *TUPLE1 *(*HIRA*) both lie within the DiGeorge chromosomal region (DGCR). The probe *TUPLE1 *is located between *CLTCL1 *and *D22S941 *and overlaps with the P250-A1 MLPA probe *HIRA *in the chromosome. The *N25 *(*D22S75*) probe lies between the P250-A1 MLPA probe *DGCR2 *and *CLTCL1 *(Figure 2).

Dual-color FISH was performed on metaphase spreads of peripheral blood lymphocytes on pre-cleaned glass slides. Co-denaturation of probes and slides was performed at 73°C for 5 min, followed by overnight hybridization at 42°C. After hybridization and washing, chromosomes and nuclei were counterstained with DAPI (Vysis, Downers Grove, IL, USA). A coverslip was then applied to each slide. Images were captured using a CCD camera (ProgRes, Jena, Germany) through an Olympus-BX51 fluorescence microscope (Olympus, Japan) with the VideoTest Fish 2.0 software package (GP Medical Technologies Ltd, Beijing, China). Generally, 10 to 20 metaphases were examined, and 30 to 50 interphase nuclei were scored for the number of signals present for each probe.

### Gene PCR and sequencing

The three different splice variants of the *TBX1 *coding regions--including exon-intron boundaries and the promoter site--were amplified in the 212 CTD patients and 139 unrelated healthy controls. Primers were designed following the corresponding genomic regions available from the GenBank database (NG_009229.10) using Primer3 software (http://frodo.wi.mit.edu/primer3/). All PCR products were sequenced on an ABI 3130 sequencer (Applied Biosystems). The sequence traces were aligned with the reference sequence using the GenBank BLAST program.

### Statistical analysis

The Hardy-Weinberg equilibrium (HWE) and allele frequencies were assessed at each single nucleotide polymorphism (SNP) locus using the *χ*^2 ^and Fisher's exact test. HWE_*P *value > 0.05 was considered as a statistically insignificant deviation from the equilibrium. The 2-sided statistical tests were considered significant with a level of *P *≤ 0.05. All statistical analyses in our study were carried out with SPSS 11.5 (SPSS Inc. Chicago, IL, USA).

## Results

### Cell culture and karyotyping

No obvious structural and numerical abnormalities were found on the metaphase spreads of the 212 patients.

### Multiplex ligation-dependent probe amplification

**Figure 3:**
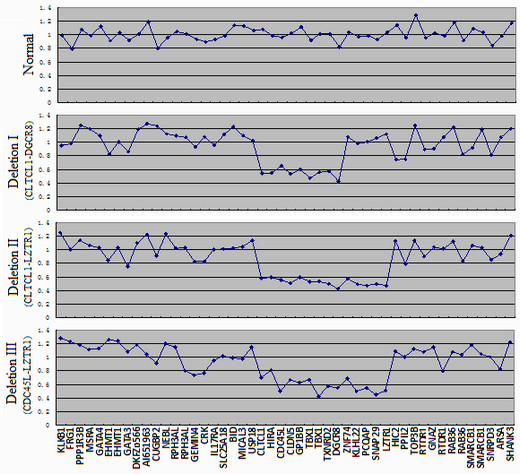
**MLPA ratio charts of the normal control and the three deletion types**. The X-axis shows the names of the probes, whereas the Y-axis indicates their ratios. All results were confirmed in two independent runs.

MLPA screening identified a loss of DNA dosage in chromosome 22q11 in 13 subjects (6.13%) out of the 212 CTD patients screened (Table 1), an incidence lower than that reported (12.8-17.8%) by authors in the West [2-5]. Of these 13 subjects, 11 were found to have an identical deletion region about 2.2-2.5 Mb in size, spanning from probe *CLTCL1 *to *LZTR1 *(LCR22-A to LCR22-D) (Figures 2 and 3). Patient NO.10D differed in that he showed a deletion region about 1.8-2.0 Mb in size, from *CDC45L *to *LZTR1 *(atypical LCR22-A to LCR22-D) (Figures 2 and 3). In patient NO.13, we found yet a smaller deletion region about 0.9-1.3 Mb in size, extending from *CLTCL1 *to *DGCR8 *(LCR22-A to LCR22-B) (Figures 2 and 3). Other probes assaying for deletions in chromosome 4p, 8p, 9p, 10p and 17p did not reveal obvious changes in the number of copies.

**Table 1:** Detailed information of the 13 CTD patients with 22q11.2 deletion

NO.	Age*	Cardiac Defects	Karyotype	Size of	FISH		TBX1
						
	(m)	Primary Diagnosis		Deletion (MLPA)	N25	TUPLE1	Sequence
T36	21	TOF	46,XY	CLTCL1-LZTR1	Del	Del	H
1E	13	TOF	46,XX	CLTCL1-LZTR1	Del	Del	H
2F	19	TOF	46,XY	CLTCL1-LZTR1	Del	Del	H
33	24	TOF	46,XX	CLTCL1-LZTR1	Del	Del	H
18	8	PA/VSD	46,XY	CLTCL1-LZTR1	Del	Del	H
1C	4	PA/VSD	46,XX	CLTCL1-LZTR1	Del	Del	H
32	23	PA/VSD	46,XY	CLTCL1-LZTR1	Del	Del	H
1I	15	PA/VSD	46,XY	CLTCL1-LZTR1	Del	Del	H
2D	23	PA/VSD	46,XX	CLTCL1-LZTR1	Del	Del	H
5	9	PA/VSD	46,XY	CLTCL1-LZTR1	Del	Del	H
10D	120	PA/VSD	46,XY	CDC45L-LZTR1	N	N	H
D2	21	DORV	46,XY	CLTCL1-LZTR1	Del	Del	H
13	8	PTA(A1)	46,XX	CLTCL1-DGCR8	Del	Del	H

Abbreviations: * age in months at the time of examination; m = months; Del = deletion; N = normal; H = haploid

### FISH

Of the 13 patients with deletions identified by MLPA, 12 were confirmed by using two FISH probes. FISH examination of their family members indicated that the deletions arose *de novo*. In patient NO.10D, with a deletion region from *CDC45L *to *LZTR1*, the signals of both probes *N25 *(*D22S75*) and *TUPLE1 *were detected in the FISH slides (data not shown).

From the combined results of MLPA and FISH, two of the deletion regions could be redefined from *N25 *(*D22S75*) to *LZTR1 *and from *N25 *(*D22S75*) to *DGCR8*. The proximal breakpoint of the deletion region from *CDC45L *to *LZTR1 *may be located on or neighboring *CDC45L*, which is at locus 0.03 Mb to *HIRA *(*TUPLE1*) (Figure 2).

### Assessment of clinical manifestation

Table 2 lists the clinical manifestations (cardiac and extracardiac anomalies) of the 13 CTD patients determined to have the 22q11.2 deletion, including seven patients with PA/VSD, four with TOF, one with PTA and one with DORV; meanwhile, none of the TGA and IAA patients had such deletions. Of the patients with deletions, eight suffered from right aortic arch (RAA) or aortopulmonary collateral arteries (APCAs), and six from extracardiac anomalies.

**Table 2:** Clinical manifestations of the 13 CTD patients with 22q11.2 deletion

NO.	Age*	Size of	Cardiac defects		Extracaridac anomalies
				
	(m)	Deletion	P.D.	S.D.	
T36	21	D22S75-LZTR1	TOF	PFO/RAA	Nil
1E	13	D22S75-LZTR1	TOF	PFO/APCA	Asymmetric cry/smile face
2F	19	D22S75-LZTR1	TOF	PFO/RAA/APCA	Nil
33	24	D22S75-LZTR1	TOF	ASD(II)	Nil
18	8	D22S75-LZTR1	PA/VSD	PDA/ASD(II)	Hypoplastic thymus
1C	4	D22S75-LZTR1	PA/VSD	PFO/APCA	Cleft palate
32	23	D22S75-LZTR1	PA/VSD	PFO/PDA/APCA	Classic dysmorphological facial features; hypoplastic thymus
1I	15	D22S75-LZTR1	PA/VSD	PDA	Nil
2D	23	D22S75-LZTR1	PA/VSD	PDA/ASD/MAPCA	Classic dysmorphological facial features
5	9	D22S75-LZTR1	PA/VSD	ASD(II)/MAPCA	inguinal hernia
10D	120	CDC45L-LZTR1	PA/VSD	PDA/ASD/APCA	Nil
D2	21	D22S75-LZTR1	DORV	VSD/PS	Nil
13	8	D22S75-DGCR8	PTA(A1)	VSD	Nil

Abbreviations: * age in months at the point of examination; m = months P.D. = primary diagnosis; S.D. = secondary diagnosis; ASD (II) = secondary atrial septal defect; APCA = aortic-pulmonary collateral arteries; MAPCA = major aortic-pulmonary collateral artery; PDA = patent ductus arteriosus; PFO = Patent Foramen Ovale; RAA = right aortic arch; PS = pulmonary stenosis.

### Gene PCR and sequencing

**Figure 4:**
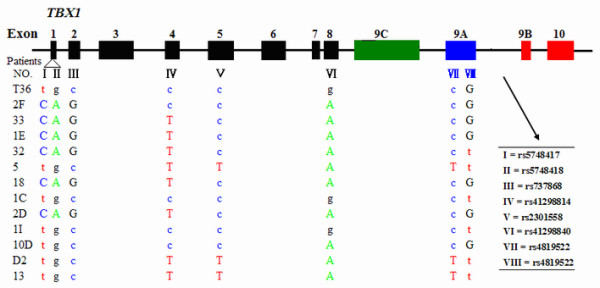
**Positions of common variants of the 13 patients with 22q11.2 deletion**. Ancestral alleles are written with small letters. The numerals from I to VIII are representative of the eight SNPs from rs5748417 to rs5746826, respectively. The corresponding identification numbers of the 13 patients with 22q11.2 deletions are listed at the left.

In the 22q11.2 deletion group, no heterozygotes of *TBX1 *were detected, which is in accordance with the partial monosomy for 22q11.2. No mutations were detected in the haploid *TBX1 *gene. However, eight different sequence variants were detected in the haploid *TBX1 *gene of the 13 del22q11 patients (Figure 4). The allele frequencies of SNPs observed in our study were similar to that observed in the Han Chinese of 1000 Genome Project Database (http://browser.1000genomes.org). All eight SNPs of HWE_*P *values were > 0.05 in the non-deletion patients and healthy controls. All of the substitutions were common variants with a minor allele frequency (MAF) of > 0.05. All variants, except for rs41298840 (c.933A > G, A311A), were detected at similar frequencies between del22q11 and non-del CTD patients (Table 3). The frequencies of polymorphisms c.933A > G were similar between the non-del22q11 CTD patients and the healthy controls, but they differed from that of the del22q11 CTD patients (*P *< 0.05) (Table 3).

**Table 3:** Allele frequencies of SNPs in del22q11 patients (n = 13), non-del patients (n = 199), controls (n = 139) and Han Chinese in 1000 Genome Project (n = 197)

SNP	^$^A F (%(n))		^$^A F (%(n))		^$^A F (%(n))		^$^A F (%(n))	
	
	del	non-del	non-del	control	del	control	control	^#^1000GPD
rs5748417								
T	53.8(7)	68.6(273)	68.6(273)	59.0(164)	53.8(7)	59.0(164)	59.0(164)	66.0(260)
C	46.2(6)	31.4(125)	31.4(125)	41.0(114)	46.2(6)	41.0(114)	41.0(114)	34.0(134)
*χ*^2^	1.261		6.600		0.136		3.427	
*P(df = 1)	0.363		0.011		0.777		0.074	
rs5748418								
G	53.8(7)	72.9(290)	72.9(290)	59.0(164)	53.8(7)	59.0(164)	59.0(164)	66.0(260)
A	46.2(6)	27.1(108)	27.1(108)	41.0(114)	46.2(6)	41.0(114)	41.0(114)	34.0(134)
*χ*^2^	2.272		14.28		0.136		3.427	
*P(df = 1)	0.203		2.0E-4		0.777		0.074	
rs737868								
G	46.2(6)	31.7(126)	31.7(126)	38.8(108)	46.2(6)	38.8(108)	38.8(108)	39.8(157)
C	53.8(7)	68.3(272)	68.3(272)	61.2(170)	53.8(7)	61.2(170)	61.2(170)	60.2(237)
*χ*^2^	1.213		3.739		0.278		0.068	
*P(df = 1)	0.364		0.059		0.772		0.081	
rs41298814								
T	61.5(8)	46.2(184)	46.2(184)	51.8(144)	61.5(8)	51.8(144)	51.8(144)	51.5(203)
C	38.5(5)	53.8(214)	53.8(214)	48.2(134)	38.5(5)	48.2(134)	48.2(134)	48.5(191)
*χ*^2^	1.185		2.031		0.472		0.005	
*P(df = 1)	0.398		0.160		0.577		1.000	
rs2301558								
T	23.1(3)	14.8(59)	14.8(59)	14.4(40)	23.1(3)	14.4(40)	14.4(40)	12.4(49)
C	76.9(10)	85.2(339)	85.2(339)	85.6(238)	76.9(10)	85.6(238)	85.6(238)	87.6(345)
*χ*^2^	0.669		0.025		0.744		0.540	
*P(df = 1)	0.426		0.912		0.744		0.489	
rs41298840								
A	76.9(10)	44.7(178)	44.7(178)	47.1(131)	76.9(10)	47.1(131)	47.1(131)	52.3(206)
G	23.1(3)	55.3(220)	55.3(220)	52.9(147)	23.1(3)	52.9(147)	52.9(147)	47.7(188)
*χ*^2^	5.275		0.379		4.416		1.737	
*P(df = 1)	0.025		0.583		0.046		0.210	
rs4819522								
C	76.9(10)	86.3(344)	86.4(344)	86.3(240)	76.9(10)	86.3(240)	86.3(240)	87.1(343)
T	23.1(3)	13.6(54)	13.6(54)	13.7(38)	23.1(3)	13.7(38)	13.7(38)	12.9(51)
*χ*^2^	0.953		0.001		0.908		0.075	
*P(df = 1)	0.403		1.000		0.404		0.818	
rs5746826								
G	53.8(7)	33.9(135)	33.9(135)	38.1(106)	53.8(7)	38.1(106)	38.1(106)	37.6(148)
T	46.2(6)	66.1(263)	66.1(263)	61.9(172)	46.2(6)	61.9(172)	61.9(172)	62.4(246)
*χ*^2^	2.211		1.264		1.292		0.022	
*P(df = 1)	0.148		0.289		0.262		0.936	

Abbreviation: ^$^A F indicates the allele frequency. * *P*-values were calculated using Fisher's exact test. ^#^the 1000 Genome Project Database.

The frequencies of rs5748417 (c.-87+242T > C) and rs5748418 (c.-87+256G > A), as shown in Table 4, were similar between the del22q11 CTD patients and controls. However, they were significantly different between the non-del22q11 CTD patients and controls (Table 3). The discrepancy may be due to the limited sample size (n = 13) of the patients with CTDs and del22q11. Further analysis of the three genotype frequencies of the two SNPs between non-del22q11 CTD patients and controls showed significant differences between the two groups at both genetic polymorphisms (Table 5).

**Table 4:** Allele frequencies of rs41298838 (c.928G > A, G310S) in the three groups

SNP	Allele Frequency (%(n))		Allele Frequency (%(n))		Allele Frequency (%(n))	
	
	del	non-del	non-del	control	del	control
G	100(13)	95.7(382)	95.7(382)	97.5(271)	100(13)	97.5(271)
A	0(0)	4.3(17)	4.3(17)	2.5(7)	0(0)	2.5(7)
*χ*^2^	1.578		1.455		0.335	
*P(df = 1)	1.000		0.292		1.000	

* *P*-values were calculated using Fisher's exact test.

**Table 5:** Genotype frequencies of rs5148417 and rs5148418 in non-del patients (n = 199), controls (n = 139) and Han Chinese in 1000 Genome Project (n = 197)

SNP	Genotype frequency (%(n))		Genotype frequency (%(n))	
	
	Non-del	control	control	1000GPD
rs5748417				
TT	48.7(97)	35.3(49)	35.3(49)	44.2(87)
TC	39.7(79)	47.5(66)	47.5(66)	43.7(86)
CC	11.6(23)	17.3(24)	17.3(24)	12.2(24)
HWE_*P*	0.801	1.000	1.000	0.975
*χ*^2^	6.522		3.337	
*P(df = 2)	0.038		0.189	
rs5748418				
GG	50.3(100)	35.3(49)	35.3(49)	44.2(87)
GA	40(80)	47.5(66)	47.5(66)	43.7(86)
AA	9.5(19)	17.3(24)	17.3(24)	12.2(24)
HWE_*P*	1.000	1.000	1.000	0.975
*χ*^2^	9.013		3.337	
*P(df = 2)	0.011		0.189	

* *P*-values were calculated using Fisher's exact test.

In addition, the non-synonymous SNP rs41298838 (c.928G > A, G310S) was found to occur at a similar frequency in both the non-del22q11 patients and healthy controls (Table 4). This result is in contrast with a previous finding by Yagi *et al*. [12], who reported it as a mutation. It is, however, in line with the study by Heike *et al.*, who used SIFT and Polyphen to predict that this non-synonymous SNP would be tolerated by the protein [17].

## Discussion

Microdeletion in the 22q11 region causes a variety of disorders, including the DiGeorge (DGS; OMIM 188400) and Velo-cardio-facial (VCFS; OMIM 192430) syndrome. Summarized as a deletion syndrome (22q11DS), it is the most frequent chromosomal microdeletion syndrome, occurring once in 4,000 live births [18]. Applying FISH with commercially available *N25 *(*D22S75*) or *TUPLE1 *probes located in the proximal commonly deleted region of 22q11.2 is a proven method for detection of the 22q11 deletion. Most of the 22q11.2 deletions can be found using FISH diagnostic probes, although this method has failed to detect deletions that are either proximal or distal to the FISH probes used [7,19,20]. Methods employing multiple genetic markers in the 22q11 region are increasingly important for the accurate identification of genomic microdeletions. MLPA analysis with multiple probes has been applied to analyze the 22q11 chromosome in detail [21], and the commercially available high-definition MLPA 22q11 kit has proven to be a sensitive tool for detecting copy number changes on the long arm of chromosome 22. In our study, in one patient (NO.10D) who tested negative for the 22q11.2 deletion using FISH probes *N25 *(*D22S75*) and *TUPLE1*, MLPA analysis actually identified an atypical deletion from *CDC45L *to *CLTCL1*.

In general, CTDs are the disorders most commonly associated with the 22q11.2 deletion syndrome [22,23]. Of the study subjects, 20% (7/35) of those with PA/VSD, 5.4% (4/74) with TOF, 33% (1/3) with PTA and 2% (1/51) with DORV were detected to have the 22q11.2 deletion. None of the patients with TGA had the 22q11.2 deletion. In the subgroup of patients presenting with the 22q11.2 deletion, 53.8% (7/13) suffered from PA/VSD, 30.8% (4/13) from TOF, and 69.2% from RAA or APCAs (Table 1 and 2). This result is in line with the findings of previous studies, which concluded that a high prevalence of the deletion is noted in patients with TOF, PA/VSD, PTA, as well as RAA and major APCAs [23,24]. Despite its commonness in patients with the 22q11.2 deletion in previous studies [25-27], none of the patients with the IAA condition examined in this study showed this deletion. This finding may be due to the small number (n = 4) of IAA patients investigated or to possible differences in the 22q11DS pathoanatomy between Western and Asian populations. Further investigation of the deletion in a larger sample size of IAA patients is necessary to clarify this issue.

To date, multiple phenotypic features and associated abnormalities have been observed in patients with the 22q11.2 deletion. However, no consistent correlation between the genotype and phenotype has been shown for this syndrome. Rauch *et al*. conjectured that conotruncal cardiac defects related to the 22q11.2 deletion are usually associated with the common 3 Mb or 1.5 Mb proximally nested deletions, both of which involve the *TBX1 *gene [11]. This study shows that all deletion types are found in the commonly deleted region, implicating the involvement of the *TBX1 *gene. However, a detailed analysis of the clinical manifestations did not reveal any genotype-phenotype correlations in the subjects of our study (Table 2).

*TBX1 *is thought to be a major candidate gene that influences the cardiac phenotype or its severity in patients carrying the 22q11.2 deletion [28]. Its dose-dependent role in heart development has been demonstrated in mouse models [29]. Therefore, screening of the *TBX1 *gene on the residual 22q11 homologous chromatid would likely indicate whether the sequence variants or mutations of the remaining *TBX1 *copy would have an impact on the phenotype, thereby revealing possible correlations between the *TBX1 *genotype and cardiac phenotype. No mutations were found in this group of patients, but eight sequence variants were found in the haploid *TBX1 *gene. When comparing the del22q11 CTD patients to the non-del CTDs patients, the frequencies of polymorphism c.933A > G (rs41298840) in the del22q11 patients significantly differed from that in the non-del22q11 CTD patients and healthy controls (*P *< 0.05). The non-synonymous SNP rs41298838 (c.928G > A, G310S), which is a known mutation [12] previously predicted to be a tolerant polymorphism [16], was found in both the non-del22q11 CTD patients and healthy controls at similar frequencies. Prior studies have found that although the TBX1^G310S ^mutation does not prevent transactivation, it does prevent the TBX1-SMAD1 interaction [30,31], which may have had an impact on the homeostasis of the cardiac progenitor cells [32]. These differences imply that the loci rs41298838 and rs41298840 may have an impact on the pathogenesis of this syndrome. However, the findings presented may be biased due to the small number of del22q11 patients studied.

The allele and genotype frequencies of SNPs rs5748417 (c.-87+242T > C) and rs5748418 (c.-87+256G > A) were significantly different between the non-del22q11 CTDs patients and controls. The alleles c.-87+242T and c.-87+256G were more frequent in the group of non-del22q11 patients with CTDs than in controls. These differences suggest the association of these SNPs in the pathogenesis of CTDs. However, further studies are needed to clarify the mechanism, especially since both of the SNPs are located within the 5'UTR intron of the *TBX1 *gene.

## Conclusions

CTDs, especially PA/VSD and TOF, are the most common disorders associated with the 22q11.2 deletion syndrome. MLPA using commercial kits is a rapid, reliable, and cost-effective method for the diagnosis of 22q11DS. Almost all del22q11 CTD patients identified in this study have a deletion region (typical or atypical) in the *TBX1 *gene. The differences in allele and genotype frequency distributions of rs5748417 and rs5748418 between non-del22q11 CTD patients and controls imply that the genetic variation in the *TBX1 *gene may be associated with CTDs. Although the *TBX1 *gene polymorphisms on the residual 22q11 homologous chromatid have not been found to be a major modifier for the pathogenesis of 22q11.2 deletion syndrome in previous studies [33,34], the fact that frequencies of the SNPs rs41298838 and rs41298840 in del22q11.2 CTD patients differed from those in the non-del CTDs patients in this study indicated that polymorphisms of the residual *TBX1 *gene may have an impact on the pathogenesis of this syndrome. Further studies with larger sample sizes are warranted to determine if this notion holds true.

## Competing interests

The authors declare that they have no competing interests.

## Authors' contributions

YJX designed the study, collected the samples, analyzed the data and drafted the manuscript. JW helped to screen the individuals and collect samples, and participated in analyzing the data. RX participated in the design of the study and helped to draft the manuscript. PJZ and XKW collected the samples. HJS and LMB helped to analyze the data. JS, QHF, and FL participated in the design of the study. KS conceived of the study, participated in its design and coordination, analyzed the data and helped draft the manuscript. All authors read and approved the final manuscript.

## Pre-publication history

The pre-publication history for this paper can be accessed here:

http://www.biomedcentral.com/1471-2350/12/169/prepub
